# RTVP-1 regulates glioma cell migration and invasion via interaction with N-WASP and hnRNPK

**DOI:** 10.18632/oncotarget.4471

**Published:** 2015-06-15

**Authors:** Amotz Ziv-Av, Nissim David Giladi, Hae Kyung Lee, Simona Cazacu, Susan Finniss, Cunli Xiang, Maor H. Pauker, Mira Barda-Saad, Laila Poisson, Chaya Brodie

**Affiliations:** ^1^ Everard and Mina Goodman Faculty of Life Sciences, Bar-Ilan University, Ramat-Gan, Israel; ^2^ Davidson Laboratory of Cell Signaling and Tumorigenesis, Hermelin Brain Tumor Center, Department of Neurosurgery, MI, USA; ^3^ Department of Public Health Sciences, Henry Ford Hospital, Detroit, MI, USA

**Keywords:** Glioblastoma, N-WASP, hnRNPK, Glioma cell migration

## Abstract

Glioblastoma (GBM) are characterized by increased invasion into the surrounding normal brain tissue. RTVP-1 is highly expressed in GBM and regulates the migration and invasion of glioma cells. To further study RTVP-1 effects we performed a pull-down assay using His-tagged RTVP-1 followed by mass spectrometry and found that RTVP-1 was associated with the actin polymerization regulator, N-WASP. This association was further validated by co-immunoprecipitation and FRET analysis. We found that RTVP-1 increased cell spreading, migration and invasion and these effects were at least partly mediated by N-WASP. Another protein which was found by the pull-down assay to interact with RTVP-1 is hnRNPK. This protein has been recently reported to associate with and to inhibit the effect of N-WASP on cell spreading. hnRNPK decreased cell migration, spreading and invasion in glioma cells. Using co-immunoprecipitation we validated the interactions of hnRNPK with N-WASP and RTVP-1 in glioma cells. In addition, we found that overexpression of RTVP-1 decreased the association of N-WASP and hnRNPK. In summary, we report that RTVP-1 regulates glioma cell spreading, migration and invasion and that these effects are mediated via interaction with N-WASP and by interfering with the inhibitory effect of hnRNPK on the function of this protein.

## INTRODUCTION

Glioblastomas (GBM), the most common and malignant primary brain tumors, are characterized by high proliferation, vascularization and invasion into the surrounding normal tissue [1-3]. The median survival of patients with GBM, following tumor resection and treatment with radiation and chemotherapy, is less than 16 months [1, 4, 5]. The poor prognosis of GBM patients is attributed to the infiltrative nature of these tumors [6-8] and to the presence of therapy resistant residual cancer stem cells [9].

Related to testis-specific, vespid and pathogenesis protein 1 (RTVP-1) was cloned from human GBM cell lines by and was termed glioma pathogenesis-related protein (GLIPR1) or RTVP-1 [10, 11]. RTVP-1 contains a putative signal peptide, a transmembrane domain and a SCP domain, which is common to other homologs of this protein [12, 13]. Our previous studies demonstrated that the expression of RTVP-1 was correlated with the degree of malignancy of astrocytic tumors [12, 13] and that it was involved in the regulation of the growth, survival and invasion of glioma cells [12]. The expression of RTVP-1 was recently reported to be increased in additional tumors including Wilm's tumors due to its hypomethylation [14] and in invasive melanoma [15]. In these tumors, silencing of RTVP-1 resulted in reduced cell migration and proliferation. In contrast, RTVP-1 expression was found to be downregulated in prostate tumors [16].

RTVP-1 expression is induced in glioma cells by the protein kinase C (PKC) isoforms, PKCα and PKCε, via phosphorylation of the transcription factor serum response factor (SRF) [17]. In addition, we recently identified RTVP-1 as a novel target of the tumor suppressor miRNA, mir-137, which inhibits the self-renewal of glioma stem cells (GSCs) and promotes their differentiation by targeting RTVP-1 and the CXCR4 pathway [18].

The WASP family consists of five members in mammalian cells, including WASP, N-WASP (neural WASP), WAVE1 (WASP family verprolin homologous protein 1), WAVE2, and WAVE3, which can be further divided into two subgroups, WASP/N-WASP and WAVEs [19]. WASP family proteins integrate multiple upstream signals to induce actin polymerization through the Arp2/3 complex, an activator of actin filament nucleation and branching [20-22]. Several lines of evidence indicate that these proteins are necessary for cell protrusive activity associated with cell migration and invasion. Moreover, the expression of the WASP family proteins has been associated with malignant phenotypes of cancer cells, including glioma [23], indicating the importance of these proteins in cancer cell migration and invasion [24].

Although our recent studies implicated RTVP-1 as a tumor promoter in GBM, the mechanisms by which this protein induces glioma cell migration and invasion are not yet understood. In this study we identified N-WASP and hnRNPK as novel interacting proteins of RTVP-1 and demonstrated that RTVP-1 induced cell spreading, migration and ECM degradation in an N-WASP dependent manner. Moreover, overexpression of RTVP-1 decreased the association of N-WASP and hnRNPK, which has been reported to inhibit N-WASP effects on cell spreading and migration.

## RESULTS

### RTVP-1 regulates the invasion and matrix degradation of glioma cells and glioma stem cells (GSCs)

We previously reported that the expression of RTVP-1 correlated with the degree of malignancy in astrocytic tumors and that overexpression of RTVP-1 induced migration [17] and invasion of glioma cells [12].

**Figure 1:**
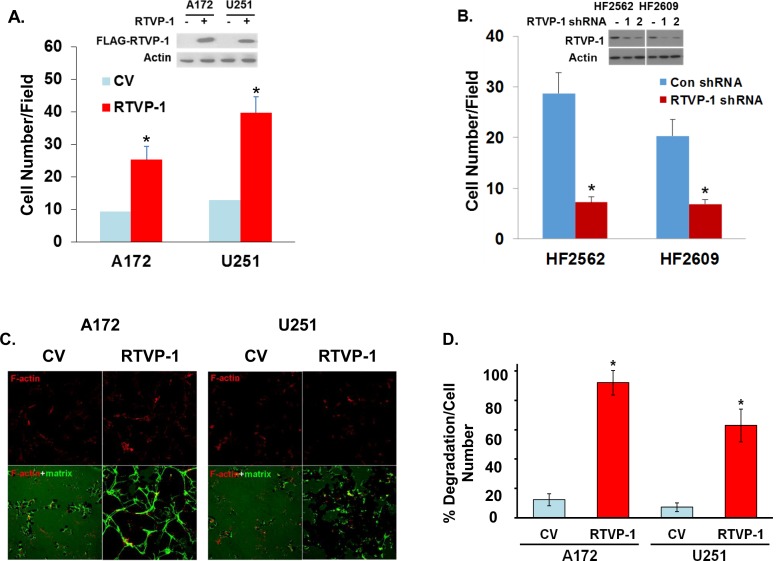

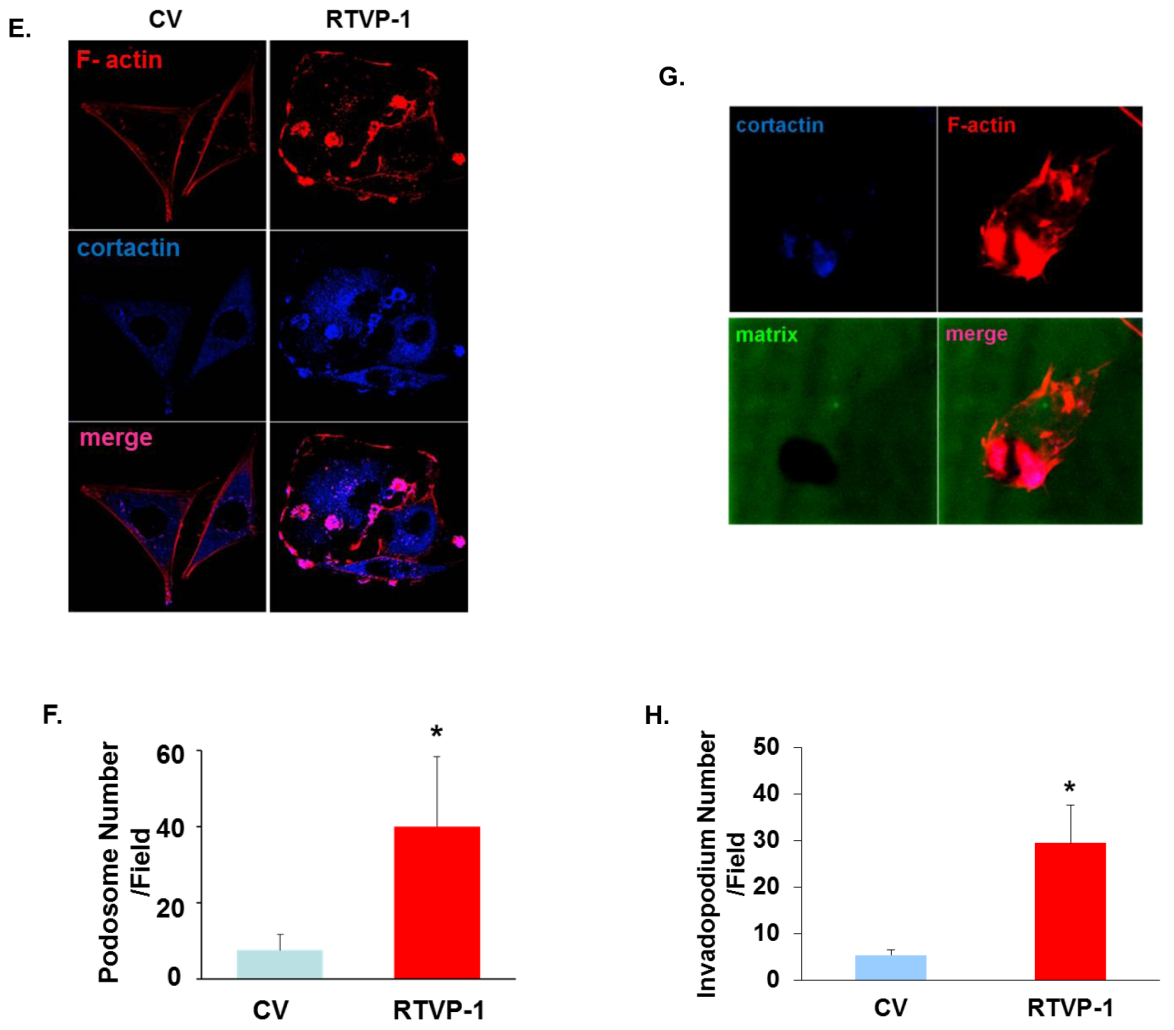
RTVP-1 promotes cell invasion and invadopodia formation A172 or U251 cells were transduced with adenovirus vectors expressing Flag-RTVP-1 or a control vector (CV) and 48 h later RTVP-1 expression was determined by Western blot analysis and cell invasion was determined using a Boyden chamber/matrigel assay **A.** Cells were plated on the top compartment of a Boyden chamber and after 8 hr of incubation, cells that traversed the matrigel-coated filters were stained and counted. Similar assays were performed for the HF2562 and HF2609 GSCs that were transduced with lentivirus vector expressing RTVP-1 or control shRNAs **B.** A172 and U251 overexpressing RTVP-1 were plated on fluorescent fibronectin/gelatin coated coverslips. Twelve hours later, the cells were fixed and stained for F-actin using TRITC-conjugated phalloidin. Representative degradation assay images of A172 and U251 cells are presented **C.** The percentage of matrix degradation per culture cell number was analyzed as described in the methods **D.** A172 cells were plated on fibronectin-coated plates and the presence of podosomes was identified by staining the cells with F-actin and anti-cortactin antibody **E.** The number of podosomes per field was determined as described in Methods **F.** Invadopodia formation in the RTVP-1 overexpressing A172 cells was identified by the co-localization of F-actin and cortactin as dark, non-fluorescent areas of degradation. A Confocal z-stack representative image shows the presence of invadopodia protrusion penetrating into the fibronectin/gelatin matrix in RTVP-1 overexpressing A172 cells; the degradation site localizes specifically at the area of F-actin and cortactin co-localization **G.** The percentage of invadopodia per cell number in CV and RTVP-1 overexpressing cells was determined in 8 different fields for triplicate wells for each cell type **H.** One representative of three similar experiments is presented. The results are presented as mean ± SE and represent three different experiments (A,B,D). *P < 0.001.

To further examine the role of RTVP-1 in glioma cell invasion we overexpressed it in glioma cell lines that express low levels of this protein. As presented in Figure 1A, overexpression of RTVP-1 in the A172 and U251 cells increased cell invasion as analyzed using the Boyden chamber assay. We also examined the role of RTVP-1 in the migration and invasion of GSCs, which have been associated with glioma cell infiltration and tumor recurrence [25, 26]. The preparation and characterization of these cells was previously reported [27]. Silencing of RTVP-1 in the HF2562 and HF2609 GSCs decreased cell invasion (Figure 1B). Similar results were previously reported for silenced U87 cells [12].

The effect of RTVP-1 on glioma cell invasion was also examined by matrix degradation assay using a fluorescent labeled gelatin. As presented in Figure 1C, overexpression of RTVP-1 in the A172 and U251 cells significantly increased gelatin degradation as compared with the control vector (CV) cells (Figures 1C and 1D) and in accordance with the results obtained for the Boyden chamber assay.

Matrix degradation has been associated with the formation of podosomes and invadopodia [28]. Podosomes are precursor structures that can mature on physiological substrates into invadopodium-type structures that exhibit a matrix degradation activity [29] and are identified by the co-localization of F-actin and cortactin [30]. To examine the effect of RTVP-1 on podosome formation in glioma cells, we employed A172 cells overexpressing RTVP-1 (Figure 1A). Cells were plated on fibronectin-coated plates and podosomes were identified by staining the cells with F-actin and anti-cortactin antibodies. As presented in Figures 1E and 1F, overexpression of RTVP-1 in the A172 cells resulted in a strong induction of podosomes in these cells compared to CV cells. To analyze the effect of RTVP-1 overexpression on invadopodia expression, cells were plated on fibronectin/gelatin-GFP and were stained for F-Actin and cortactin. Invadopodia were identified as structures stained for both F-actin and cortactin that were also able to degrade the fluorescent matrix (Figure 1G). The number of the invadopodia was significantly higher in A172 cells overexpressing RTVP-1 as compared to CV cells (Figure 1H).

### RTVP-1 is associated with N-WASP

**Figure 2:**
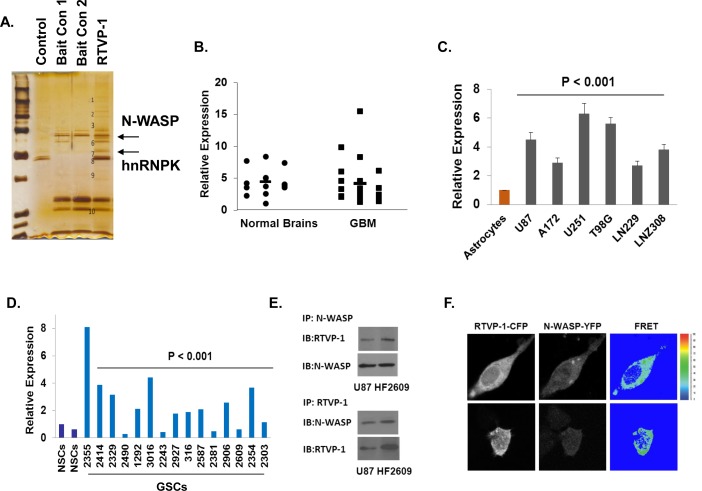
Interaction of RTVP-1 and N-WASP in glioma cells His-tag affinity pull-down assay was employed as a screening assay for identifying RTVP-1 interacting proteins. The interacting complexes were resolved and stained for further analysis. N-WASP and hnRNPK were two of the pull-down complexes identified with MassSpec analysis **A.** Total RNA was extracted from normal brains and GBM specimens and the expression of N-WASP was determined using real-time PCR. Data from individual human tissues are presented with the median and interquartile range noted. Age adjusted *t*-test, *P* = 0.001. Results were normalized relative to the levels of S12 mRNA and are presented relative to a reference sample **B.** The expression of N-WASP was also examined in human astrocytes and glioma cell lines **C.** and in human neural stem cells (NSCs) and fifteen cultures of glioma stem cells GSCs **D.** using real-time PCR. U87 glioma cells and the HF2609 GSCs were analyzed for the association of RTVP-1 and N-WASP using reciprocal co-immunoprecipitation **E.** FRET analysis using RTVP-1 tagged to CFP and N-WASP tagged to YFP was performed. The two plasmids were co-transfected into U87 cells and 24 h later the cells were fixed and FRET efficiency was determined as described in the methods **F.** One representative of three similar experiments is presented. *P* < 0.001.

To elucidate the mechanism underlying the effects of RTVP-1 on migration and invasion by RTVP-1 we performed a pull-down assay using a His-tagged RTVP-1 in U87 glioma cell lysates followed by a mass spectrometry analysis (Figure 2A). We identified the key actin regulator protein N-WASP [31] and heterogeneous nuclear ribonucleoprotein K (hnRNPK) [32] as potential interacting proteins of RTVP-1. We first examined the expression of N-WASP in normal brain and GBM specimens and found no significant differences in the expression of this protein (Figure 2B). In contrast, we found that N-WASP expression was increased in glioma cell lines compared with normal human astrocytes (Figure 2C) and in glioma stem cells (GSCs) compared with neural stem cells (NSCs) (Figure 2D). We then analyzed the interaction of RTVP-1 with N-WASP since this protein plays a major role in actin polymerization and cell migration [33]. Using reciprocal immunoprecipitation analyses, we confirmed the interaction of RTVP-1 and N-WASP in the U87 cells and the HF2609 GSCs (Figure 2E). To further validate this interaction we performed FRET analysis using RTVP-1 tagged to CFP and N-WASP tagged to YFP. The two plasmids were co-transfected into U87 cells and 24 h later the cells were fixed and FRET efficiency was determined as described in the methods. As presented in Figure 2F, RTVP-1 and N-WASP showed FRET efficiency of 33.43 + 2.72%, suggesting a direct interaction of these two proteins in glioma cells.

### N-WASP mediates RTVP-1 effects on cell spreading and migration of glioma cells

**Figure 3:**
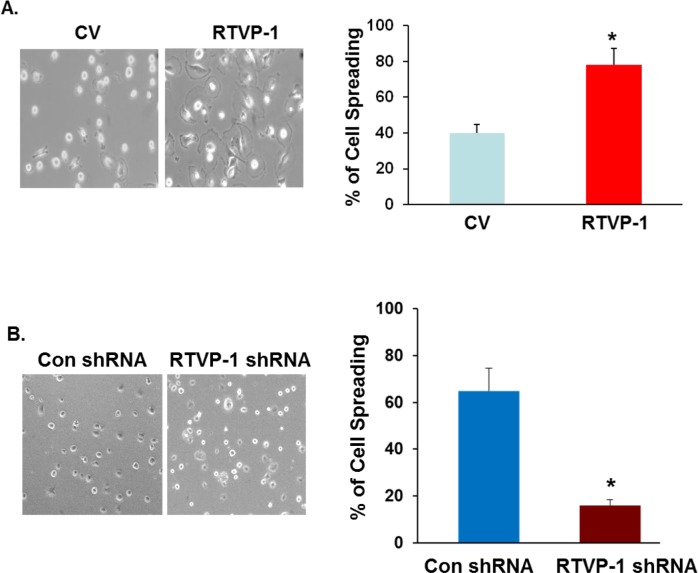

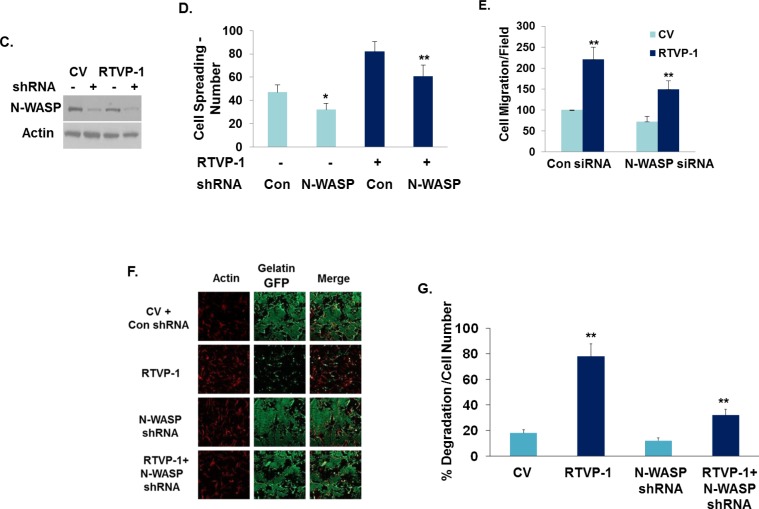
The effect of RTVP-1 on glioma cell spreading and migration is mediated by N-WASP U87 cells transfected with control vector or RTVP-1 **A.** or A172 cells were silenced for RTVP-1 **B.** for 3 days were examined for cell spreading. The figures are from one representative out of three similar experiments. The results are presented as mean ± SE and represent three different experiments (A, B). *p < 0.05. A172 cells overexpressing RTVP-1 were transfected with N-WASP shRNA, or with a non-specific shRNA as a transfection control and the expression of N-WASP was analyzed using Western blot analysis **C.** Spreading assays **D.**, transwell migration **E.** and ECM degradation assay (F, G) were performed as described in the methods. One representative of three similar experiments is presented. *p < 0.05; **p < 0.0001.

Cell adhesion and motility depend on the interactions of cells and extracellular matrix substrates. When plated onto matrix coated surfaces, cells first flatten and deform extensively as they spread [34]. Cell spreading is driven by actin polymerization, which is regulated by actin nucleation machinery involving the Arp2/3 complex [35] that is activated by the WASP protein family [36]. We next examined the effects of RTVP-1 on glioma cell spreading and the role of N-WASP in this process and found that overexpression of RTVP-1 in the A172 cells enhanced cell spreading (Figure 3A), whereas silencing of RTVP-1 in the U87 cells inhibited this process (Figure 3B).

We then studied the interaction of RTVP-1 and N-WASP in glioma cell functions. For these studies RTVP-1 was overexpressed in A172 cells that were silenced for N-WASP (Figure 3C), and cell spreading, migration and ECM degradation were determined. A decrease of ∼30% was observed in the spreading of RTVP-1 overexpressing cells that were silenced for N-WASP as compared to cells that were not silenced for this protein (Figure 3D). Similarly, silencing of N-WASP also abrogated the increased cell migration (Figure 3E) and fibronectin/gelatin degradation induced by overexpression of RTVP-1 in A172 cells (Figures 3F, 3G). Thus, the effects of RTVP-1 on glioma cell migration, spreading and ECM degradation were at least partially mediated by its interaction with N-WASP.

### hnRNPK is an interacting protein of RTVP-1 that is downregulated in GBM and inhibits cell migration

**Figure 4:**
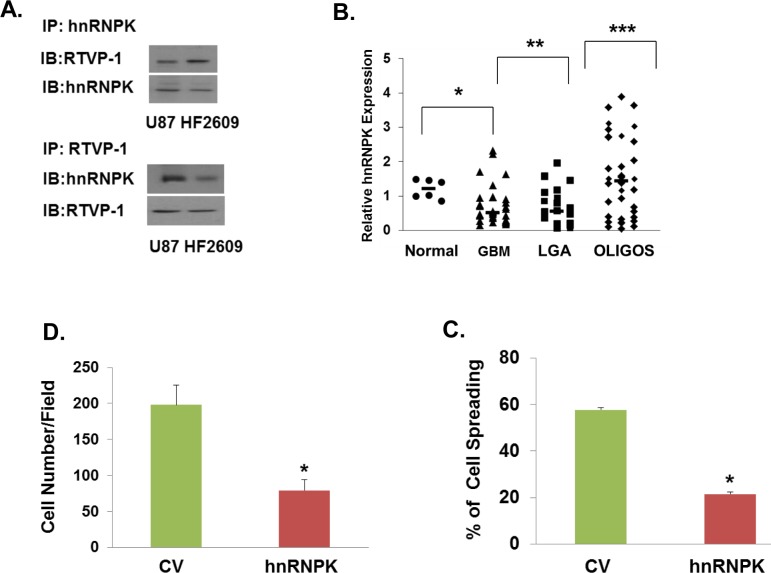

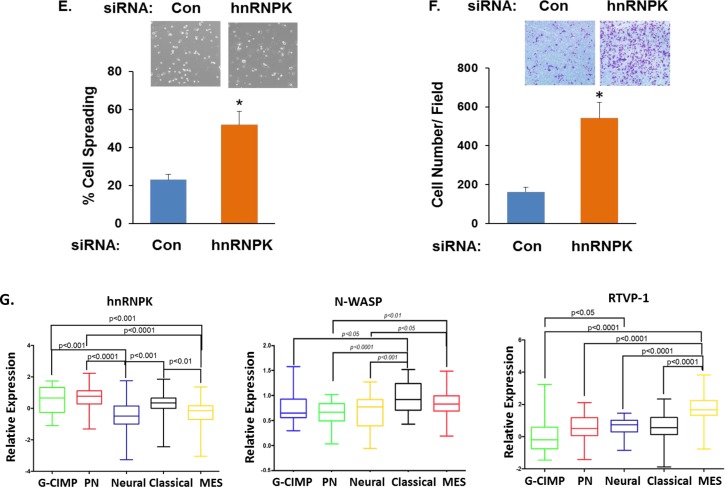
hnRNPK inhibits cell migration and its interaction with RTVP-1 decreases its association with N-WASP The association of RTVP-1 and hnRNPK was analyzed in U87 cells using reciprocal co-immunoprecipitation with either anti-N-WASP or RTVP-1 antibodies **A.** Total RNA was extracted from normal brains (NB) and astrocytic tumor specimens and the expression of hnRNPK was determined using real-time PCR **B.** Data from individual human tissues are presented with the median and interquartile range noted. Age adjusted *t*-test, *P* = 0.001. Results were normalized relative to the levels of S12 mRNA and are presented relative to a reference sample **B.** A172 cells were transfected with a vector overexpressing hnRNPK, or with a control vector (CV). Cell spreading assays **C.** and transwell migration assays **D.** were performed as described in the methods. U87 cells were transfected with hnRNPK siRNA or with a non-specific siRNA as a transfection control. Cell spreading assays **E.** and transwell migration assays **F.** were performed. The means ± S.E. from three independent experiments are shown as relative spreading after normalization to that in mock transfected cells. *p < 0.05. hnRNPK, N-WASP and RTVP-1 expression was examined in different subtypes of GBM. The gene expression plot presenting five GBM subtypes of 496 primary GBM specimens from TCGA showing differential hnRNPK gene expression **G.** ANOVA F-test is presented.

Another protein that was identified as a putative interacting protein of RTVP-1 is hnRNPK. Inhibition of hnRNPK was reported to increase cell spreading [37]. Moreover, hnRNPK was reported to directly interact with N-WASP in the periphery of spreading cells and overexpression of hnRNPK reversed the formation of filopodia induced by N-WASP [33]. Since hnRNPK was identified as a potential interacting protein of RTVP-1 using the pull-down assay, we first validated the interaction of these two proteins using reciprocal co-immunoprecipitation in glioma cells and GSCs (Figure 4A). We then examined the expression and function of hnRNPK in glioma cells. We found that hnRNPK was highly expressed in normal brain and its expression was lower in astrocytic tumors in a tumor grade manner and in an inverse pattern to that of RTVP-1 (Figure 4B). Interestingly, hnRNPK expression was higher in oligodendroglioma compared with astrocytic tumors.

We then examined the effects of hnRNPK on glioma cell spreading, migration and invasion. We found that overexpression of hnRNPK inhibited glioma cell spreading (Figure 4C) and migration (Figure 4D), whereas silencing of hnRNPK induced the opposite effect (Figures 4E and 4F). Thus, hnRNPK is expressed in low levels in glioma and its low expression is associated with increased migration and invasion.

Recent studies have identified five GBM subtypes, which are classified based on their transcriptional signatures into proneural, G-CIMP, neural, classical and mesenchymal subtypes [38, 39]. These subtypes have distinct differential genetic alterations, molecular signature and cellular phenotypes and in particular, the mesenchymal subtype of GBM is characterized by an increased level of infiltration and poor prognosis. Since hnRNPK inhibits cell migration in glioma cells, we examined its expression in the different GBM specimens. Using the TCGA data portal [26], we analyzed the relative expression of hnRNPK in the five GBM subtypes and found that its mean expression was significantly higher in tumors that are classified as the proneural and C-CIMP and the lowest in the mesenchymal subtypes (Figure 4G). In contrast, the expression of both N-WASP and RTVP-1 was lower in the proneural GBM compared to the mesenchymal subtype (Figure 4G).

### RTVP-1 decreases the association of N-WASP and hnRNPK

**Figure 5:**
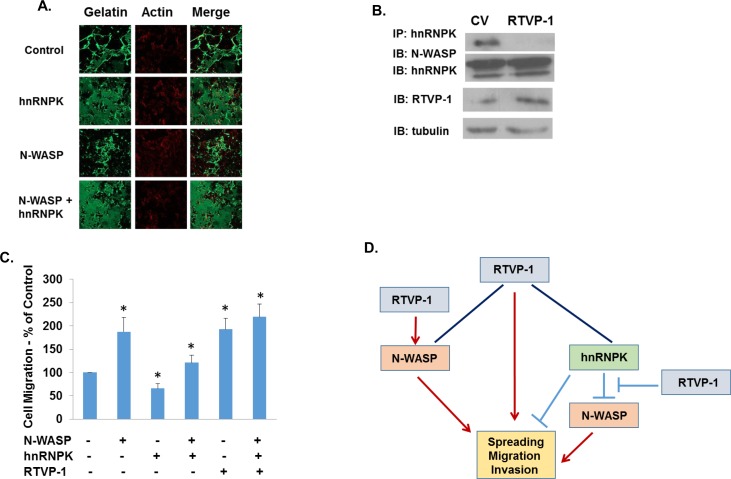
RTVP-1 inhibits the interaction of N-WASP and hnRNPK in glioma cells Fluorescent gelatin degradation assays were performed using cells transfected with hnRNPK, N-WASP or a combination of both **A.** Lysates were prepared from U87 cells (CV cells and RTVP-1-overexpressing cells) and immunoprecipitation was performed with anti-hnRNPK antibody. The immunoprecipitates (IP) and aliquots of the lysates were analyzed by Western blotting with anti-N-WASP, anti-hnRNPK, and anti-RTVP-1 antibodies **B.** Cell transwell migration assay was performed in A172 cells overexpressing N-WASP, hnRNPK and combination of both with and without RTVP-1 **C.** One representative of three similar experiments is presented. *P* < 0.001. A model demonstrating that N-WASP mediates some of RTVP-1 effects on cell spreading, migration and invasion and that RTVP-1 promotes N-WASP effects by decreasing the association of hnRNPK and N-WASP (Figure 5D).

The association of hnRNPK with N-WASP has been reported to inhibit N-WASP effects on cell spreading. We therefore examined if this interference occurs also in glioma cells. As presented in Figure 5A, overexpression of hnRNPK decreased the gelatin degradation of the U87 glioma cells, whereas overexpression of N-WASP induced the opposite effect. Overexpression of hnRNPK inhibited the ECM degradation induced by N-WASP, similar to its inhibitory effects on N-WASP-induced cell spreading [33]. Since both RTVP-1 and hnRNPK are associated with N-WASP but have opposite effects on cell spreading and ECM degradation, we hypothesized that RTVP-1 may interfere with the association of hnRNPK and N-WASP. To test this hypothesis we examined the association of N-WASP and hnRNPK in the presence or absence of RTVP-1. We found that overexpression of RTVP-1 significantly reduced the association of N-WASP and hnRNPK (Figure 5B) and abrogated the inhibitory effect of hnRNPK on N-WASP-induced cell migration when co-expressed in the A172 glioma cells (Figure 5C). Thus, our data indicate that N-WASP mediates some of RTVP-1 effects on cell spreading, migration and invasion and that RTVP-1 promotes N-WASP effects by decreasing the inhibitory impact of hnRNPK (Figure 5D).

## DISCUSSION

GBM represent 70% of all malignant primary brain tumors [1]. The infiltration of tumor cells into the surrounding brain tissue and the presence of treatment resistant GSCs are the main cause of tumor recurrence and poor prognosis of GBM patients [40]. In this study we explored the mechanisms by which RTVP-1 induced glioma cell migration and invasion and identified the key actin regulator N-WASP as a novel interacting protein and a mediator of RTVP-1 effects.

We found that overexpression of RTVP-1 in A172 cells, which express low levels of this protein, induced cell invasion as indicated by both Boyden chamber and matrix degradation assays. We next examined the effect of silencing of RTVP-1 on the migration of GSCs, which are implicated in therapeutic resistance, tumor invasion and recurrence [38, 39] and found that silencing of RTVP-1 in these cells resulted in decreased migration and invasion.

We recently identified RTVP-1 as a novel target of the tumor suppressor miR-137. We demonstrated that miR-137 was downregulated in GBM due to promoter hypermethylation and that miR-137 inhibited GSC self-renewal and promoted the differentiation of these cells by targeting RTVP-1 which downregulated CXCR4 [18]. The role of RTVP-1 in GSC migration and invasion, as shown here, is in agreement with its function in GSC self-renewal and further marks RTVP-1 as an attractive therapeutic target for the eradication of GSCs.

Since RTVP-1 increases cell migration and induces MMP-2 activity [12], we next performed fluorescent gelatin degradation assay and found a significant higher degree of degradation in response to RTVP-1 overexpression. Recent reports demonstrated that in metastatic melanoma cells elevated RTVP-1 levels are correlated with increased invasive potential, and that silencing of RTVP-1 reduces cell migration, invasion and proliferation [15]. Thus, RTVP-1 appears to play a major role in tumor migration and invasion.

Invasive tumor cells extend protrusions from their ventral surface into the surrounding matrix, such as podosomes, which result in degradation of the ECM at discrete foci [41]. In parallel to the induction of cell invasion and ECM degradation by RTVP-1 we also found that RTVP-1 induced the formation of podosomes and invadopodia in glioma cells. In recent years it has become clear that invadopodia have an important role in the invasion of glioma [30], and several proteins which are involved in the formation of invadopodia in glioma cells have been further explored as potential therapeutic targets such as synaptojanin [42], diaphanous-related formin 1 [43], the small GTPase RhoG [44] and CRN2 [45].

There is a strong correlation between elevated MMP levels, such as MMP-2 and MMP-9 and tumor cell invasiveness in glioma [46]. The fact that these ECM-degrading enzymes are secreted from invadopodia [30] and our previous finding that RTVP-1 induces activation of MMP-2 [12] are in agreement with the current observation that RTVP-1 induces the formation of invadopodia and the degradation of ECM.

To further explore the molecular mechanism underlying the induction of glioma invasion by RTVP-1 we performed a pull-down assay using His-tagged RTVP-1 and glioma cell lysates followed by a mass spectrometry analysis and identified the key actin regulator N-WASP as a novel interacting protein of RTVP-1. We further validated this interaction using reciprocal co-immunoprecipitation and FRET analysis. We found that although the expression of N-WASP was not significantly increased in GBM specimens compared with normal brains, it was higher in glioma cell lines and GSCs as compared with human astrocytes and GSCs, respectively, similar to the results that were recently obtained for RTVP-1.

Actin polymerization mediated by the N-WASP-Arp2/3 pathway was shown to be involved in invadopodia formation in various cellular systems [30]. Moreover, the overexpression of deleted or mutant forms of the N-WASP activator, cortactin, that prevent the binding of specific partners such as Arp2/3 complex, F-actin or N-WASP, significantly decreases invadopodia formation and ECM degradation [47], showing that N-WASP is necessary for the invasion process. In gliomas it was recently shown that N-WASP is a key mediator of low oxygen-induced brain invasion [23].

Cell spreading is driven by actin nucleation machinery involving Arp2/3 complex [35], which is activated by the WASP protein family [36]. We therefore examined the effect of RTVP-1 and N-WASP on cell spreading. We found that overexpression of either N-WASP or RTVP-1 increased cell spreading while silencing of RTVP-1 inhibited this process. We further demonstrated the role of N-WASP in RTVP-1 effects by silencing the expression of N-WASP in RTVP-1 overexpressing cells. A significant decrease in the effect of RTVP-1 on cell spreading, migration and ECM degradation was observed in N-WASP silenced cells, indicating that N-WASP is at least partially mediating RTVP-1 effects.

Another interacting protein of RTVP-1 that was identified in the pull-down assay was hnRNPK. Heterogeneous nuclear ribonucleoproteins (hnRNPs) are a family of RNA binding proteins which have a central role in a variety of cellular processes including DNA repair, telomere biogenesis, cell signaling and the regulation of gene expression at both the transcriptional and translational levels [48]. hnRNPK is involved in cell spreading and is localized to structures called spreading initiation center (SIC) which are similar but distinct from the more mature focal adhesions. Indeed, inhibition of hnRNPK by antibodies increases cell spreading [37] and silencing of hnRNPK in differentiated N2A neuroblastoma cells increases the number and length of their neurites [49]. Moreover, hnRNPK inhibits cell spreading and filopodia formation by a direct association with N-WASP [33]. We validated the interaction of hnRNPK and RTVP-1 by co-immunoprecipitation and showed that hnRNPK was associated with N-WASP also in glioma cells.

We found that hnRNPK was highly expressed in normal brain and its expression was decreased in astrocytic tumors in a tumor grade-dependent manner which is an inverse pattern to that of RTVP-1 [12]. Similarly, the expression of hnRNPK was the lowest in the mesenchymal GBM subtype, in contrast to the expression of RTVP-1. Overexpression of hnRNPK induced an inhibitory effect on cell spreading and migration of glioma cells whereas its silencing induced opposite effects. Thus, low levels of hnRNPK in glioma cells was associated with increased cell migration and invasion. Interestingly, the expression of hnRNPK is increased in a variety of tumors and in some studies hnRNPK was reported to promote tumor metastasis [50]. The reason for the differences in expression and functions of hnRNPK in different cell systems is not understood; however, the function of hnRNPK is dependent on its subcellular localization, i.e. cytoplasmic *vs*. nuclear [51, 52], and tyrosine phosphorylation [53] which may be cell-and tumor dependent. In a recent study it was shown that the nuclear level of hnRNPK is reduced in progressive murine fibrosarcoma compared to regressive tumors [54].

Based on the interaction of both RTVP-1 and hnRNPK with N-WASP and their opposite effects on glioma cell spreading, migration and invasion we speculated that RTVP-1 might compete with hnRNPK for the association with N-WASP. Indeed, overexpression of RTVP-1 decreased the association of N-WASP with hnRNPK suggesting that RTVP-1 may activate N-WASP by decreasing its inhibitory association with hnRNPK. Our conclusion was further validated by demonstrating that overexpression of RTVP-1 was able to abrogate the inhibitory effect of hnRNPK on N-WASP-induced glioma cell migration.

In summary, we report that RTVP-1 regulates glioma cell spreading, migration and invasion and that these effects are mediated via interaction with N-WASP and by interfering with the inhibitory effect of hnRNPK on the function of N-WASP. Thus, RTVP-1 is a potential therapeutic target for the inhibition of glioma cell and GSC migration and invasion.

## MATERIALS AND METHODS

### Materials

Antibodies for hnRNPK, His tag, N-WASP and cortactin were obtained from Santa Cruz (Santa-Cruz, CA). Oregon-green gelatin was purchased from Sigma Aldrich. Phalloidin-TRITC was obtained from Invitrogen (Carlsbad, CA).

### Human tissue specimens

Frozen human non-tumor brain tissues and human GBM specimens were obtained from the Department of Neurosurgery at Henry Ford Hospital. All human materials were used in accordance with the policies of the Henry Ford Hospital institutional review board (IRB # 6399).

### Cell cultures

The glioma cell lines A172, U251 and U87 were obtained from the American Type Culture Collection (Manassas, VA). Cells were maintained as previously described [55].

### Generation of primary GSC cultures

All human materials were used in accordance with the policies of the Henry Ford Hospital IRB. The generation, characterization and maintenance of GSCs were previously described [18, 27, 55, 56]. Briefly, fresh GBM specimens were processed and spheroids were maintained in neurosphere medium (DMEM-F12 1/1, supplemented with bFGF (20 ng/ml) and EGF (20 ng/ml)). The GSCs were examined for self–renewal, neural differentiation and for their tumorigenic potential in nude mice.

### Transfection and transduction of glioma cells

Small interfering RNA (siRNA) duplexes were synthesized and purified by Dharmacon (Lafayette, CO). siRNA pools consisted of four siRNA duplexes for RTVP-1, N-WASP or hnRNPK duplexes were employed.

Transfection of glioma cells and GSCs HF2562 and HF2609 with RTVP-1, N-WASP or hnRNPK siRNA duplexes (SMARTpool, Thermo Scientific, Lafayette, CO) was performed by siIMPORTER (Millipore, Billerica, MA) following the manufacturer's recommendations.

Lentivirus vectors (System Biosciences, Mountain View, CA) expressing RTVP-1 and shRNAs for RTVP-1, N-WASP and hnRNPK were packaged and used to transduce the cells according to the manufacturer's protocol. The medium was then replaced with fresh medium, and the cells were used 72 h post infection. For each experiments, we employed two different shRNA constructs that gave similar results.

The AdEasy system was kindly provided by Dr. Vogelstein (The Johns Hopkins University School of Medicine, Baltimore, MD) [57]. Adenoviruses expressing RTVP-1 were prepared as previously described [58]. Cells were incubated with 5 multiplicity of infection of the recombinant adenovirus vectors for 1 h. The medium was then replaced with fresh medium and the cells were used 24 to 48 h post infection.

### Transwell migration assay

Transwell chambers (BD Biosciences, San Jose, CA) were used according to the manufacturer's protocol and as previously described [59]. Briefly, cells were harvested, resuspended in serum-free medium, and then transferred to the transwell chambers (25,000 cells per well). The chambers were then incubated for 3 h in culture medium with 10% FBS in the bottom chambers before analysis. The migrating cells on the lower surface were fixed and stained with 0.05% crystal violet and stained cells were counted under a microscope. Wound healing assay was done using culture-inserts purchased from Ibidi, LLC (Verona, WI) according to the manufacturer's protocol.

### *In vitro* invasion assay

Boyden chamber invasion assays were performed as previously described [12].

### Cell spreading assay

Cells were trypsinized and incubated with gentle agitation in serum-free medium at 37°C for 1 h. The cells were then plated on fibronectin-coated cover slips and allowed to spread for the indicated times (about 20 min). Multiple fields were imaged and spreading cells were defined as cells that were completely flattened and no longer had the white ring that is characteristic of floating cells.

### Preparation of His tag RTVP-1 protein, Affinity pull-down assay and protein identification

Recombinant His-tagged RTVP-1 protein was produced and purified as described previously. After the washing process, the interacting proteins were eluted for analysis in-gel followed by mass spectrometry. Briefly, His-tagged RTVP-1 was immobilized on metal chelate (cobalt) agarose beads and then incubated with U87 cell lysates. The beads were then washed in washing buffer, and the bound proteins were eluted and size-fractioned by SDS/PAGE. Gels were stained with SimplyBlue SafeStain (Invitrogen) for band excision and mass spectrometry. Analysis of excised in-gel digested bands was carried out by using a LC-nano MS/MS spectrometer (NextGen Sciences, Ann Arbor, MI). The sequences of individual peptides were identified by using the Mascot algorithm to search and correlate the MS/MS spectra with amino acid sequences in the protein database.

### Immunofluorescence staining and podosome formation

For immunofluorescence staining the cells were fixed in 4% paraformaldehyde for 20 min and permeabilized with wash solution (0.1% Triton X-100, 1% bovine serum albumin in PBS) for 20 min. Cells were incubated with rabbit anti-cortactin polyclonal antibody (1:300) for 45 min, washed three times with PBS and incubated with Cy5 anti-rabbit antibody for 1 h. Coverslips were mounted on slides using anti-fade solution.

For the identification of podosomes, cells were incubated with rabbit anti-cortactin polyclonal antibody (1:300) for 45 min, washed three times with PBS and incubated with Cy5 anti-rabbit antibody for 1 h. For F-actin staining, cells were incubated with TRITC-conjugated phalloidin (1:200) for 20 min. Coverslips were mounted on slides using anti-fade solution. For quantifying matrix degradation, images of 10 fields/10mm² per slide were acquired using eight-bit 512×512 pixel confocal Zeiss LSM510 microscope and AIM software. The percentage of degraded matrix per slide was analyzed using ImageJ software.

### Extracellular matrix degradation assay

Fluorescently labeled fibronectin/gelatin-coated coverslips were prepared as described recently [55, 60]. Briefly, coverslips were coated with Oregon green 488-conjugated fibronectin/gelatin mixture (Sigma Chemical Co., St. Louis, MO) + 2% sucrose, cross-linked for 15 min in 0.5% glutaraldehyde in PBS, and incubated in 5 mg/ml NaBH_4_ in PBS for 3 min. After washing with DMEM at 37°C, cells were plated on coated coverslips in DMEM and incubated for 17 h.

### Western blot analysis

Western blot analysis was performed as described [61]. Equal loading was verified using an anti-β-actin antibody.

### Real-time quantitative PCR analysis

Total RNA was extracted using RNeasy midi kit according to the manufacturer's instructions (Qiagen, Valencia, CA). Reverse transcription reaction was carried out using 2 μg total RNA as described for the RT-PCR analysis. A primer optimization step was tested for each set of primers to determine the optimal primer concentrations. Once the optimal primer concentrations were determined, primers, 25 μl of 2× SYBR Green Master Mix (Invitrogen, Carlsbad, CA), and 30 to 100 ng cDNA samples were resuspended in a total volume of 50 μl PCR amplification solution. The following primers were used: hnRNPK forward, and S12 forward, TGCTGGAGGTGTAATGGACG; S12 reverse, CAAGCACACAAAGATGGGCT.

### FRET analysis

FRET was measured by the donor-sensitized acceptor fluorescence technique. Three sets of filters were used, one optimized for donor fluorescence (Exc. 468 nm, Em. 475-505 nm), a second for acceptor fluorescence (Exc. 514 nm, Em. 530LP), and the third for FRET (Exc. 468 nm, Em. 530LP). FRET was corrected as described below and the FRET efficiency was determined. To measure FRET, three images were acquired for each set of measurements: YFP excitation/YFP emission image (YFP channel); CFP excitation/CFP emission image (CFP channel); and CFP excitation/YFP emission image (FRET channel). The latter is a raw uncorrected FRET image, which contains two non-FRET components: (a) the bleed-through of the CFP emission into the YFP detection channel and (b) the cross-excitation of YFP by the CFP excitation laser.

To calculate and eliminate the non-FRET components, a set of reference images was acquired from single-labeled CFP- or YFP-expressing cells for each set of acquisition parameters used to obtain the FRET experimental images. Using a pixel-by-pixel comparison of the intensity values within the CFP channel images to the FRET channel images obtained from the CFP-only expressing cells, a calibration curve was derived, which expressed the level of CFP bleed-through into the FRET channel as a function of the CFP fluorescence in the CFP channel. Such curve was plotted for each set of acquisition parameters used in the FRET measurements.

Similarly, calibration curves expressing the level of YFP cross-excitation by the CFP excitation laser as a function of YFP fluorescence were derived using YFP channel and FRET channel images obtained from the YFP-only expressing cells. Subsequently, based on the pixel-by-pixel comparison of the FRET channel versus the CFP channel and the FRET channel versus the YFP channel, images were obtained from the double-labeled cells and using the calibration curves.

The non-FRET components were calculated and subtracted from each raw FRET image yielding corrected FRET images. The FRET efficiency was calculated on a pixel-by-pixel basis using the following equation: FRETeff=FRETcorr/(FRETcorr + CFP)x100%, where FRET corr is the pixel intensity in the corrected FRET image and CFP is the intensity of the corresponding pixel in the CFP channel image. To increase the reliability of the calculations and to prevent low-level noise from distorting the calculated ratio, pixels below 50 intensity units and saturated pixels were excluded from the calculations and their intensities were set to zero.

### Statistical analysis of TCGA data

The results are presented as the mean values ± SD. The data of patient specimens are presented graphically with median and interquartile range noted. Data were analyzed using analysis of variance or a Student's t-test. Correlation was assessed with the Pearson's correlation coefficient and tested against a correlation of zero (no correlation). The squared Pearson coefficient, or coefficient of determination (R2) for a single predictor regression, is given on the scatter plots. Kaplan-Meier analysis was used to produce survival curves with differences tested between groups by the log-rank test. Data were analyzed on a log 2 scale as appropriate.

### Statistical analysis

The results are presented as the mean values ± SD. The data of patient specimens are presented graphically with median and interquartile range noted. Data were analyzed using analysis of variance or a Student's t-test with correction for data sets with unequal variances. An age-adjusted t-test was taken from a linear model including age as a covariate. Correlation was assessed using Pearson's correlation coefficient and tested against a correlation of zero (no correlation). The squared Pearson coefficient, or coefficient of determination (R^2^) for a single predictor regression, is given on the scatter plots. Kaplan-Meier analysis was used to produce survival curves with differences tested between groups by the log-rank test. Data were analyzed on a log 2 scale as appropriate.
